# Deep Neural Network-Aided Histopathological Analysis of Myocardial Injury

**DOI:** 10.3389/fcvm.2021.724183

**Published:** 2022-01-10

**Authors:** Yiping Jiao, Jie Yuan, Oluwatofunmi Modupeoluwa Sodimu, Yong Qiang, Yichen Ding

**Affiliations:** ^1^Key Laboratory of Measurement and Control of CSE, Ministry of Education, School of Automation, Southeast University, Nanjing, China; ^2^Department of Bioengineering, Erik Jonsson School of Engineering and Computer Science, The University of Texas at Dallas, Richardson, TX, United States; ^3^Hamon Center for Regenerative Science and Medicine, The University of Texas Southwestern Medical Center, Dallas, TX, United States

**Keywords:** deep neural network (DNN), heart failure, endomyocardial biopsy, histopathology (HPE), computer-aided diagnosis (CAD)

## Abstract

Deep neural networks have become the mainstream approach for analyzing and interpreting histology images. In this study, we established and validated an interpretable DNN model to assess endomyocardial biopsy (EMB) data of patients with myocardial injury. Deep learning models were used to extract features and classify EMB histopathological images of heart failure cases diagnosed with either ischemic cardiomyopathy or idiopathic dilated cardiomyopathy and non-failing cases (organ donors without a history of heart failure). We utilized the gradient-weighted class activation mapping (Grad-CAM) technique to emphasize injured regions, providing an entry point to assess the dominant morphology in the process of a comprehensive evaluation. To visualize clustered regions of interest (ROI), we utilized uniform manifold approximation and projection (UMAP) embedding for dimension reduction. We further implemented a multi-model ensemble mechanism to improve the quantitative metric (area under the receiver operating characteristic curve, AUC) to 0.985 and 0.992 on ROI-level and case-level, respectively, outperforming the achievement of 0.971 ± 0.017 and 0.981 ± 0.020 based on the sub-models. Collectively, this new methodology provides a robust and interpretive framework to explore local histopathological patterns, facilitating the automatic and high-throughput quantification of cardiac EMB analysis.

## Introduction

Heart failure is a major public health issue with a prevalence of over 23 million worldwide (1). Along with endomyocardial biopsy (EMB), non-invasive imaging methods such as an echocardiogram and magnetic resonance imaging (MRI) are the most common tools for diagnosing myocarditis, heart transplant rejection, and chemotherapy-induced injury (2, 3). EMB is a useful but invasive modality for making a definite diagnosis in diseases that are often difficult to diagnose by imaging modality alone. However, current grading methods in assessing histological patterns of myocardial injury are labor-intensive, error-prone, and suffer from a high inter-rater variability (4). Thus, a robust and reproducible method for the quantitative analysis of EMB is urgently needed.

Machine learning methods have been an integral part of biomedical research (5, 6) and clinical work (7, 8), having the great potential to overcome the intra- and inter-observer variability (9, 10) and to improve diagnostic accuracy and efficiency (11). These computational models are based on algorithms that can extract features from clinical data (12). Compared to traditional machine learning methods that rely on expert knowledge to transform raw image data into features (e.g., texture, statistics, and wavelet transform coefficients) (13, 14), deep neural networks (DNN) can achieve better accuracy without defining features explicitly. In the field of cardiovascular diseases, deep learning has been widely implemented for image classification and segmentation in multiple modalities, including echocardiography, coronary artery calcium scoring, coronary computed tomography angiography, single-photon emission computed tomography, positron emission tomography, magnetic resonance imaging, and optical coherence tomography (8, 15–17).

Histopathological image analysis remains the gold standard for diagnosing many diseases. DNN has been proven to be a powerful approach in the analysis of histopathological images of tumor specimens; DNN can predict tumor metastasis (11) and has been shown to be useful for tumor grading (18) and tumor microenvironment analysis (19). While some researchers reported the implementation of DNN into EMB images, the interpretability of DNN output remains challenging.

This study established and validated an interpretable DNN model to assess EMB data of patients with myocardial injury. To extract and classify representative features of myocardial injury on local histological patterns, we adapted a well-established VGG-19 model (20). We then applied the other two methods, gradient-weighted class activation mapping (Grad-CAM) (21) and uniform manifold approximation and projection (UMAP) embedding method (22), to elucidate the model outputs and visualize the intermediate features made by the VGG-19 model. Moreover, we introduced a novel multi-model ensemble strategy to minimize the intra- and inter-observer variability of random dataset partition. Collectively, our method enables automatic quantification of EMB images related to cardiomyopathy, creating a series of visualizable archives for efficient and accurate pathological inspection and providing new insight into cardiac image analysis enhanced by machine learning.

## Materials and Methods

### Data Collection

We used a publicly available dataset provided in a previous study for DNN model development and evaluation (23). Hematoxylin and eosin (H&E) stained EMB tissue samples were collected from left ventricles of 209 patients registered at the University of Pennsylvania, including 94 end-stage heart failure cases diagnosed with either ischemic cardiomyopathy (*n* = 51) or idiopathic dilated cardiomyopathy (*n* = 43), and 115 non-failure cases (23). The non-failure cases were organ donors without a history of heart failure; the hearts were not used for transplantation. Each case included 11 random ROIs within the myocardium, corresponding to 11 specific areas of 50 × 50 μm^2^, i.e., 250 × 250 pixels.

In machine learning, a dataset is usually divided as a training set, validation set, and held-out test set, used for model training, model tuning, and evaluation, respectively. In this study, the aforementioned dataset was divided on case-level into the development set (104 cases, corresponding to 1144 ROIs) for training and validation or multi-model ensemble, and the held-out test set (105 cases, corresponding to 1155 ROIs), removing the crosstalk between development and test sets.

A single model was trained on the well-established development set (23), including 70 cases (770 ROIs) for training and 34 cases (374 ROIs) for validation (Table 1). We further employed a multi-model ensemble mechanism using the five-fold-based cross-validation (see **Cross-validation for multi-model ensemble**), where 10 models were trained and integrated to improve accuracy.

**Table 1:** Number of cases used in the model development and validation.

**Subset partition**		**Non-failure**	**Failure**
Individual model (Development set)	Training	38	32
	Validation	19	15
Multi-model ensemble (Development set)	Fold-1	12	10
	Fold-2	12	10
	Fold-3	11	9
	Fold-4	11	9
	Fold-5	11	9
Held-out test set		58	47

*Each case includes 11 regions of interest (ROIs)*.

### Deep Neural Network for Myocardium Assessment

**Figure 1:**
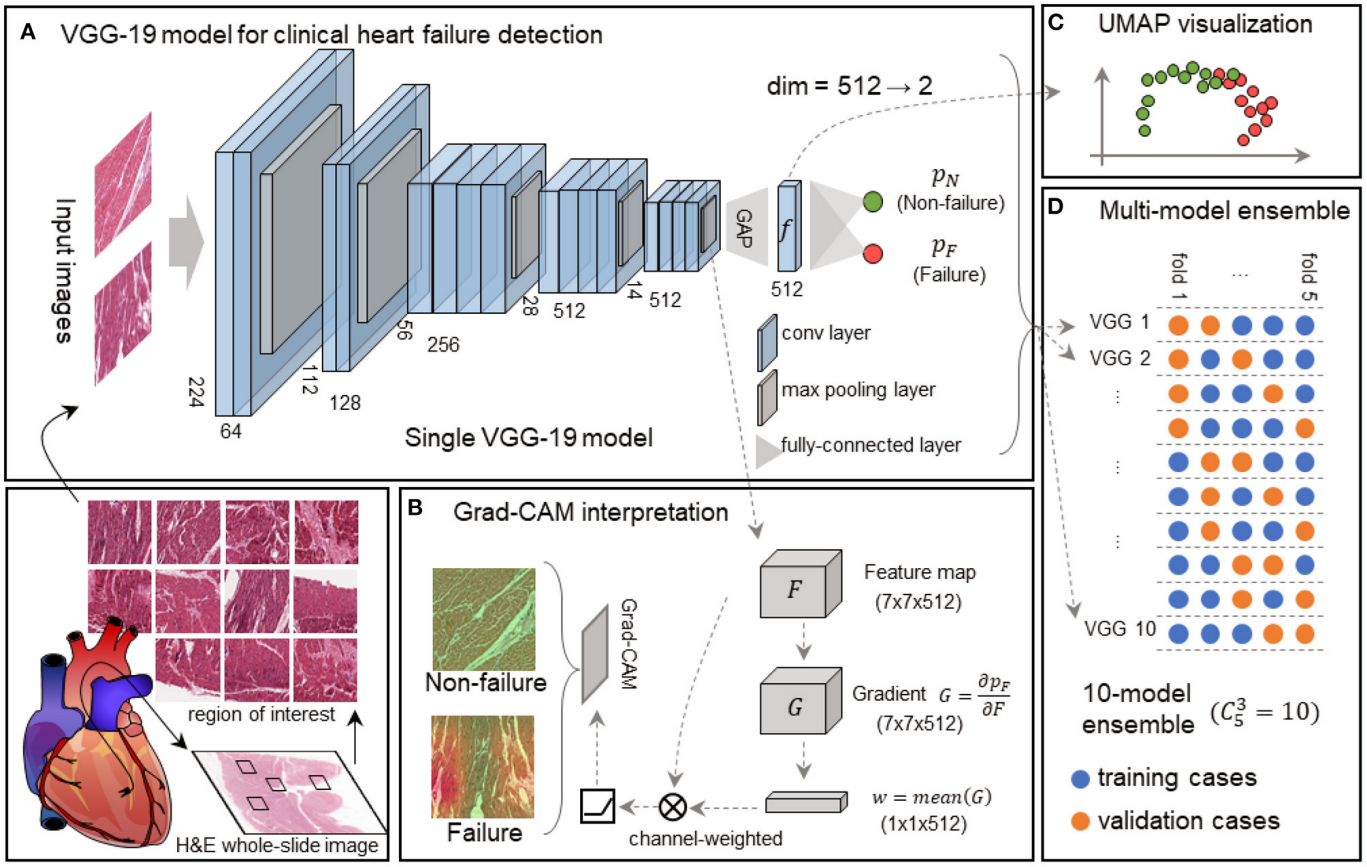
Framework of the interpretable VGG-19 model with Grad-CAM and UMAP embedding for heart failure detection. **(A)** A schematic diagram highlights the basic pipeline from the myocardial section to digital prediction. The feature extractor of this single VGG-19 network includes 16 convolutional layers and 5 max-pooling layers, and the classifier is composed of a global average pooling (GAP) layer and a classification layer. This model is trained to discriminate failure and non-failure regions of interest (ROIs) without explicit pathological patterns. dim, dimension. **(B)** Grad-CAM generates attention maps for failure and non-failure cases, following the convolution and rectified linear unit (ReLU). **(C)** UMAP in the deep feature space converts 512-dimension features to 2-dimension visualization, elucidating the model outputs and intermediate features made by the VGG-19 model. **(D)** The 10-model ensembling is generated by a 5-fold cross-validation manner, and each fold is used for sub-model validation four times.

We used VGG-19 network (20) to analyze EMB images. VGG-19 network has been widely used in computational pathology (24, 25). The first part of the model was composed of 16 convolutional layers and five max-pooling layers as the feature extractor. The rest of the model was composed of a global average pooling (GAP) layer and a classification layer with two nodes (Figure 1A). In comparison to the original VGG-19 network, our model is light-weighted and compatible with other parts of our framework (see **Regional and feature interpretability**). In our classification task, an input image was processed by all the layers and turned into a probability distribution (*p*_*F*_ or *p*_*N*_) among all the classes in the output layer (Figure 1A). From an overall view, the model receives input image of shape 224 (width) × 224 (height) × 3 (channels), and outputs a Bernoulli distribution, where the *p*_*F*_ activation gives the possibility that the input image is acquired from a heart failure patient. The entire model can be automatically optimized by minimizing the discrepancy between the network activation and desired output for end-to-end training.

### Regional and Feature Interpretability

We employed the Grad-CAM method (21) to highlight the potentially injured regions projected for interpretation and implemented the UMAP embedding to visualize inter-sample similarity generated by our VGG-19 model.

The 7 × 7 × 512 tensor was obtained from the feature extractor conveys abstract semantic information to the Grad-CAM to generate an attention map (Figure 1B) and UMAP to reduce the dimension (Figure 1C). We defined the output tensor from the last pooling layer in the VGG-19 model as *F*, and the predicted failure probability as *p*_*F*_. Grad-CAM calculated the gradient as $G=\frac{\partial p_{F}}{\partial F}$, followed by a global-average pooling as a convolutional kernel *w*. Given an input size of 224 × 224 × 3, the output size of *F*, *G*, and *w* were 7 × 7 × 512, 7 × 7 × 512, and 1 × 1 × 512, respectively. The final attention map (*A*) is determined as follows:


$$\begin{array}{l} A=ReLU\left(F⊗w\right), \end{array}$$

where ⊗ represents convolutional operation, and ReLU is defined as:


$$\begin{array}{l} ReLU\left(x\right)=x\;if\;x>0\;else\;0. \end{array}$$

The attention map highlights the regions in an input image that are mostly responsible for prediction. Thus, it provides a way to interpret the decision made by the deep neural network.

In addition to the region-based interpretation, we utilized the UMAP embedding technique to reveal the discrepancy between failing samples and non-failing samples in the feature space. We gathered deep features immediately before the final classification layer. Each input image corresponds to a deep feature vector with a length of 512. Next, we calculated the pair-wise Euclidean distances among all the ROIs, and the distance matrix was processed by UMAP (22), resulting in a 2-D embedding for each ROI. The embedding could be visualized as a scatter plot (Figure 1C), reflecting the spatial relationships among samples.

### Cross-Validation for Multi-Model Ensemble

Cross-validation is widely used to evaluate the performance of machine learning models reliably in small datasets. The dataset is generally partitioned to *K* portions, where each portion is known as a “fold.” Based on the partition, we usually use arbitrary *m* = *K*−1 portions to train a model and evaluate its performance on the rest of one portion. For this reason, we further introduced this multi-model ensemble mechanism based on cross-validation to mimic multiple human experts for consultation in pathology and minimize the randomity caused by dataset partition. We partitioned the development set into *K* = 5 subsets on case-level (Table 1) and used *m* = 3 out of *K* = 5 subsets to train a sub-model and the rest for validation at each time. All the $C_{K}^{m}=10$ models were independently trained with the identical protocol above. This allowed us to generate $C_{K-1}^{K-m-1}=4$ independent predictions to validate the training process prior to the model deployment on the held-out test set (Figure 1D). The final decision from the multi-model ensemble relied on the averaging results to eliminate the discrepancy among individual models. The whole strategy of the multi-model ensemble mechanism is similar to the pathology consultation in which experience and knowledge vary from different experts, providing a comprehensive insight into ambiguous cases.

## Experiments and Results

**Figure 2:**
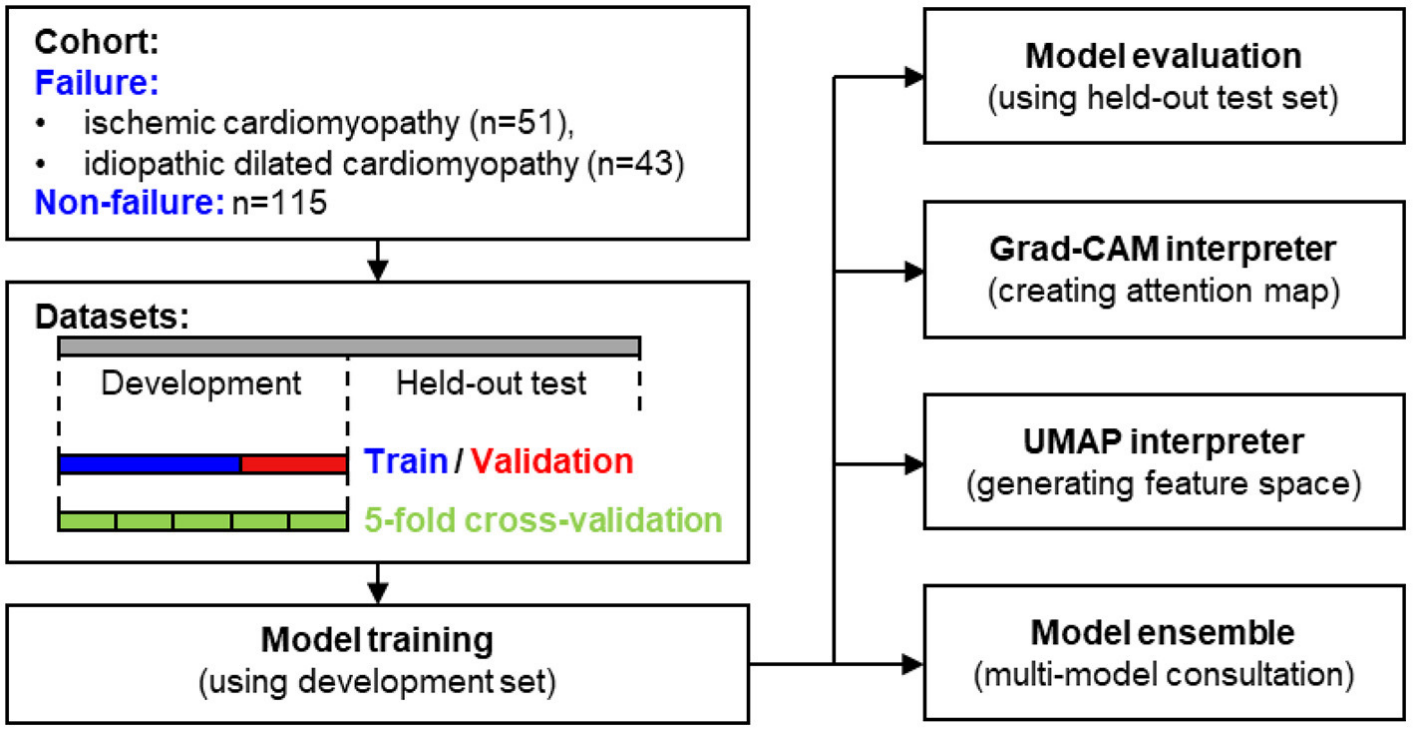
The flowchart of the study.

The study flowchart is shown in Figure 2. Given the study cohort, image samples, and partitioned datasets, both individual model and multi-model ensemble were trained and evaluated for clinical heart failure detection. Furthermore, we integrated model interpretation techniques, including Grad-CAM-based regional visualization and UMAP-based feature space visualization, to generate positive predictions for specific local histological patterns such as fibrous infiltration and the enlarged myocardial cell nuclei.

### Training and Validation of an Individual Model

**Figure 3:**
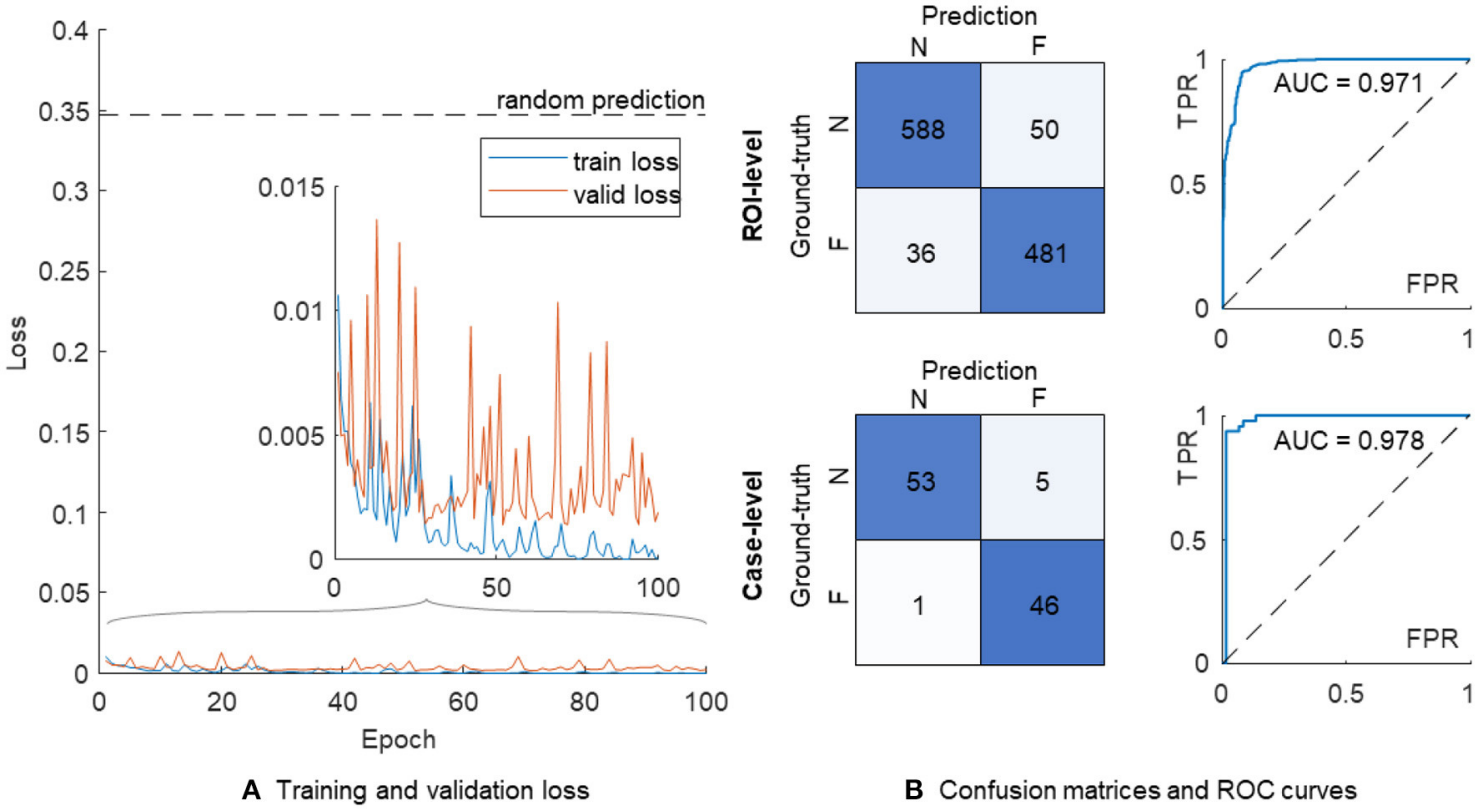
The training process and performance of a single VGG-19 model. **(A)** Training (in blue) and validation (in orange) losses. **(B)** The confusion matrices and ROC curves are shown on the ROI-level (top) and case-level (bottom), respectively. N, non-failure; F, failure; ROC, receiver operating characteristic; AUC, area under ROC curve; FPR, false positive rate; TPR, true positive rate.

We established an individual VGG-19 model on 770 training ROIs from 38 non-failure and 32 failure cases. The model was validated using 19 non-failure and 15 failure cases in 100 epochs (Table 1). The model was initialized with parameters pre-trained on ImageNet (20). The trainable parameters were then optimized by an Adam optimizer (26) (with a constant learning rate of 10^−4^) to minimize cross-entropy loss:


$$\begin{array}{l} L=\frac{1}{\left|C\right|}{\sum^{\text{​}}}_{c\in C}-y_{c}\log({\hat{y}}_{c}), \end{array}$$

where *C* is the label set of the dataset, *y*_*c*_∈{0, 1} is the one-hot encoded label of a sample, and ŷ_*c*_ is the corresponding prediction obtained from the output layer of the network. In response to appearance variation among numerous ROIs, we adopted data augmentation techniques, including random 224-pixel cropping, horizontal and vertical flipping, and stain augmentation (27) in the training process. Both training and validation losses were calculated and recorded at the end of each training period. The optimal network parameters with the lowest validation loss were retrieved for the assessment on the held-out test set (Figure 3A).

The confusion matrix on the ROI level showed 588 and 481 correct classifications in 638 non-failure and 517 failure cases, respectively (Figure 3B). We further used the proportion of positive predictions of heart failure among 11 ROIs in each patient as the aggregated risk score on the case level, achieving 53 and 46 correct classifications out of 58 non-failure and 47 failure cases, respectively. The AUC values were 0.971 and 0.978 on the ROI- and case-level, respectively.

### Grad-CAM-Based Local Visualization

**Figure 4:**
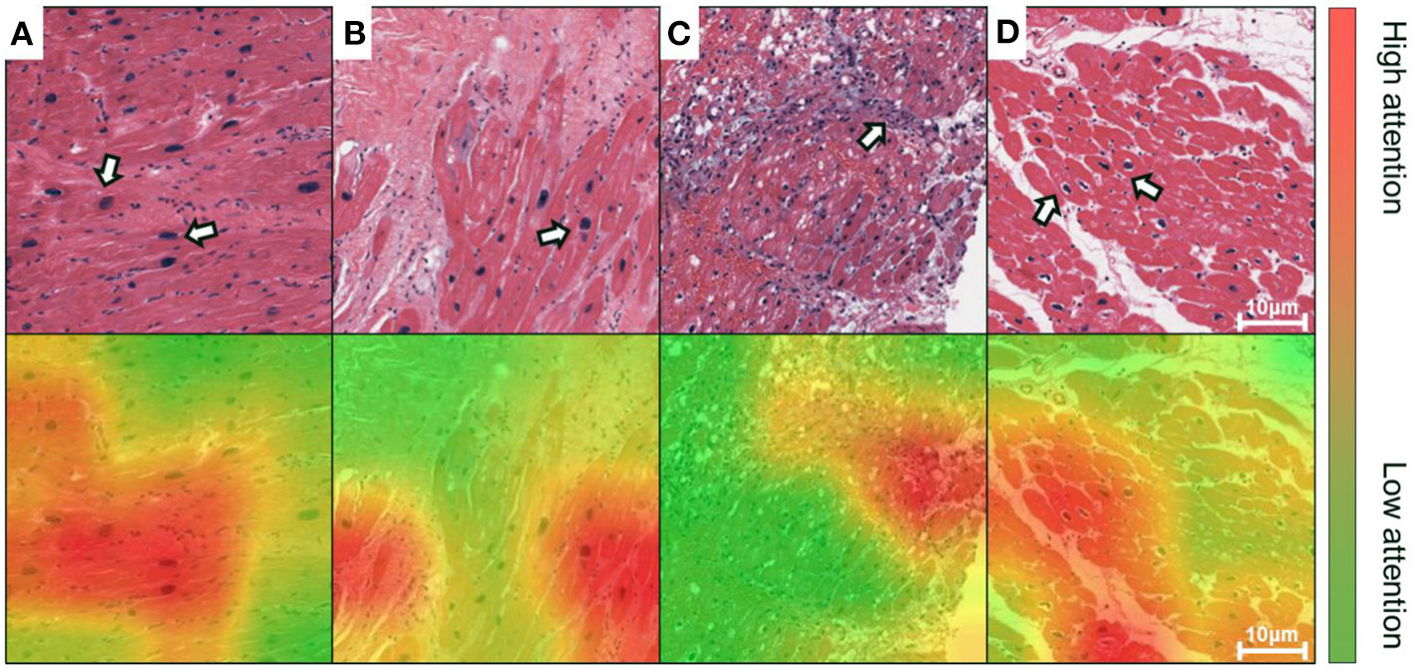
Grad-CAM generates attention maps to visualize the dominant morphology on endomyocardial biopsy (EMB) images. The attention maps are visualized as overlaid heatmaps on top of the H&E stained histopathological images. All suspected injured regions, including **(A,B)** enlarged nuclei, **(C)** inflammatory infiltration, and **(D)** perinuclear vacuolation, are indicated by arrows in the top panel or labeled as high attention in red in the bottom, whereas the other regions with low attention are in green. Scale bars: 10 μm.

We implemented the Grad-CAM to map the positive confidence to corresponding locations in the raw EMB images, demonstrating that the predictive capability of the VGG-19 model is correlated with dominant morphology such as enlarged nuclei (Figures 4A,B), inflammatory infiltration (Figure 4C), and perinuclear vacuolation (Figure 4D). All the morphologies above were labeled as high attention (arrows in the top panel and corresponding regions in the bottom) in contrast to the medium or low attention in surroundings. The results implied that this deep model could recognize specific morphological patterns in the local area on the ROI. The Grad-CAM provided a straightforward visualization method to interpret the complicated features from the DNN model, guiding us to concentrate on delicate inspection in high attention regions. We further highlighted that Grad-CAM automatically generated attention maps in accordance with pathologies, indicating that this model learned a certain level of pathological knowledge bypassing explicitly defined pathological patterns. Collectively, the attention map visualization improved the creditability and interpretability of the deep models.

### UMAP Embedding-Based Global Feature Space Visualization

**Figure 5:**
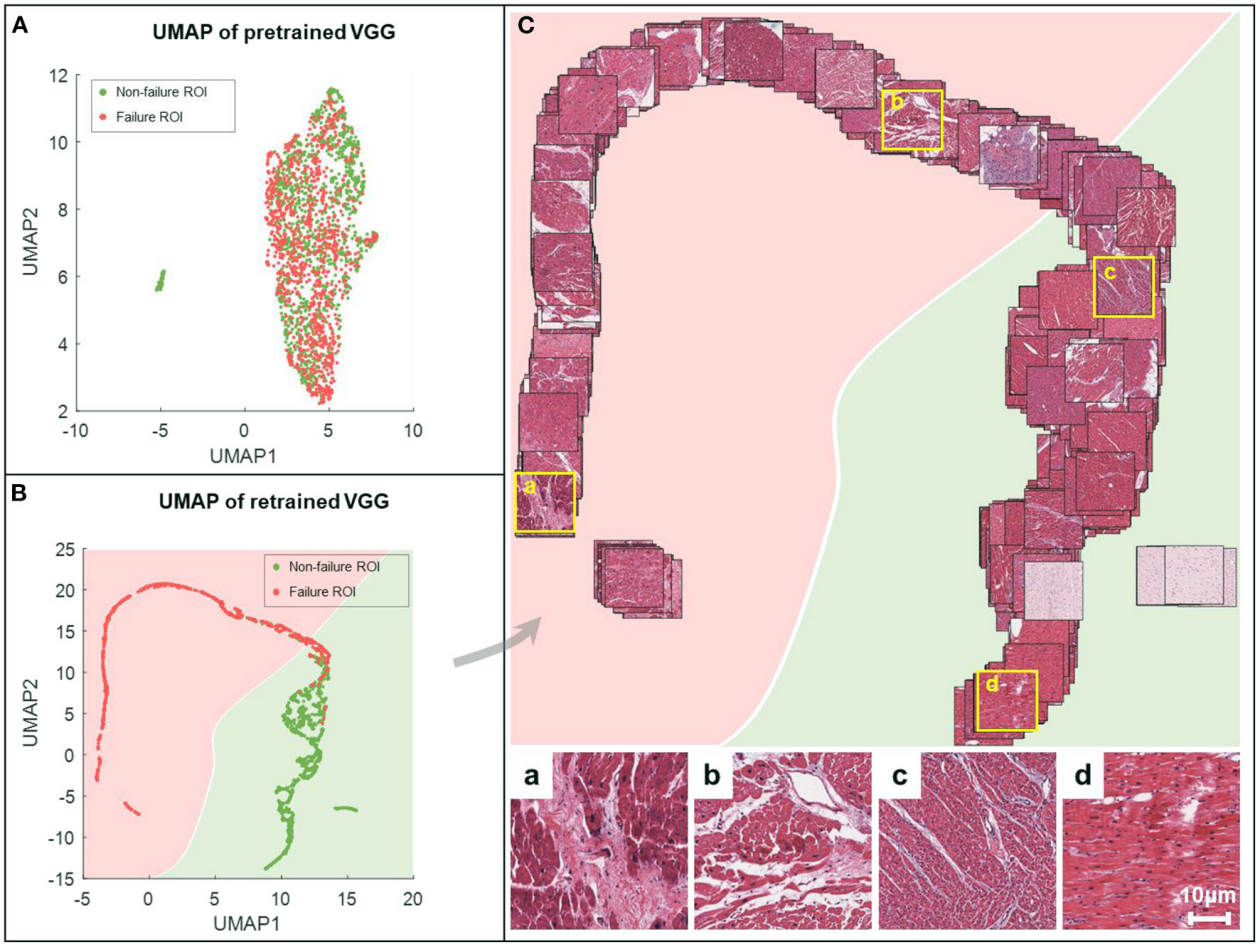
UMAP embeddings of the deep features obtained from VGG-19 networks. All the images are processed by the feature extractor, resulting in features of length 512. These features are then reduced to 2-dimension (2-D) via UMAP (red for failure label, green for non-failure label) for the characterization of spatial relationships on the ROI-level. The 2-D UMAP embeddings are given for pretrained VGG **(A)** and retrained VGG models **(B)**. **(C)** Corresponding EMB images are projected onto the UMAP coordinates for the sake of visualization. For better visual effect, 20% of samples are randomly selected. Representative examples are shown in the bottom, where the first two (a,b) are failure samples, and the others (c,d) are non-failure samples.

The UMAP embedding method reduced the dimensionality of the intermediate tensor at the end of the feature extractor in the VGG-19 model, following a GAP layer. We converted all images in the development set and the held-out test set to 512 × 1 column vectors and used the UMAP method to generate 2-D embeddings of all the images. The failure and non-failure samples were mixed together and could not be divided if processed by dimension reduction in the feature space of the original VGG-19 model (Figure 5A). In comparison, our retrained model generated a clear boundary between two groups showing regular distribution in the deep feature space (Figure 5B). This suggests that the feature extractor is effectively re-modulated in the heart failure detection task.

We further projected all ROIs to the corresponding coordinates in the UMAP space, providing an intuitive distribution of non-failure (in green) and failure (in pink) images (Figure 5C). Compared to the failure group, the non-failure images were correlated with densely arranged myocardium cells. Our results indicated that the VGG-19 model was still sensitive to specific applications, and retraining was mandatory to improve the generalization capability.

### Multi-Model Ensemble

**Figure 6:**
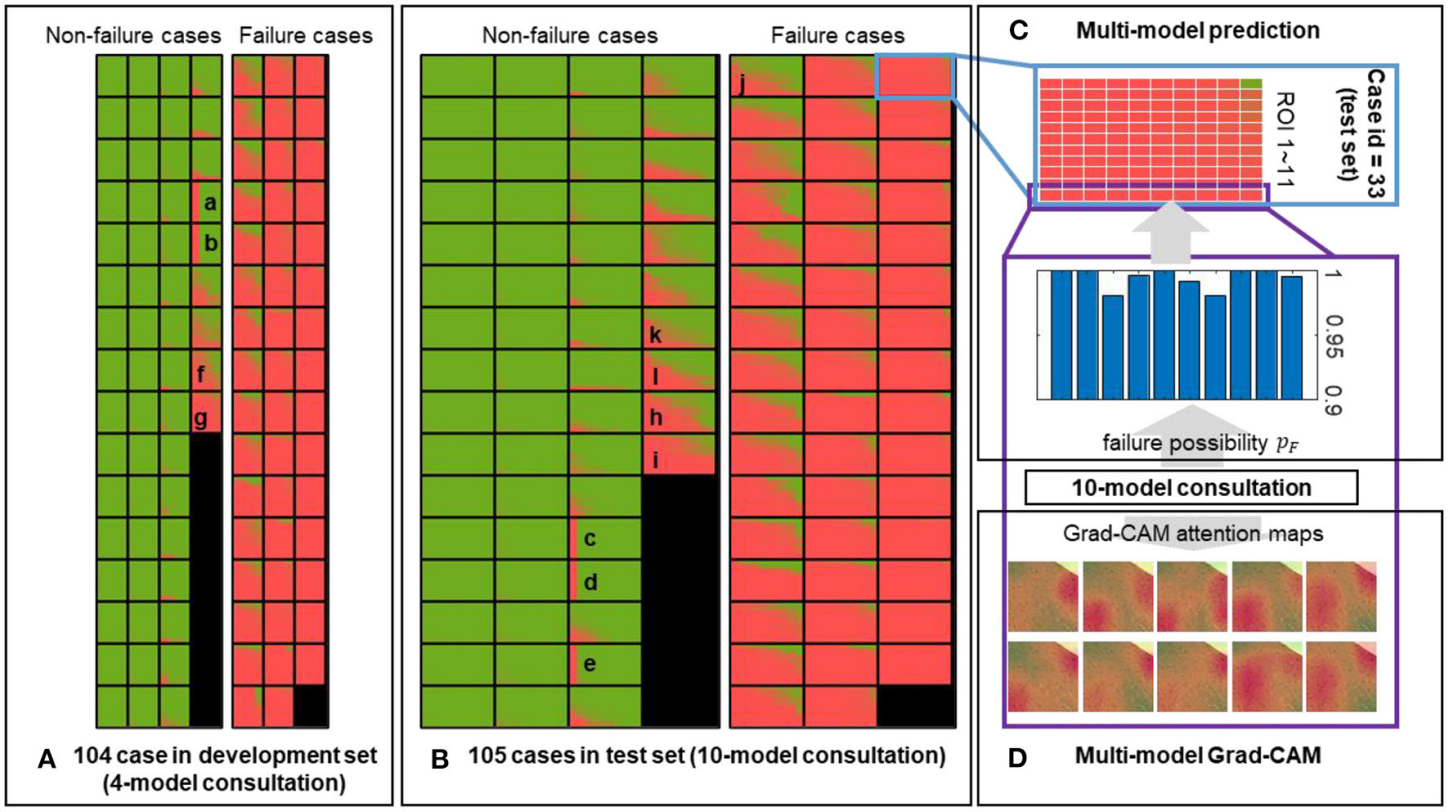
Predictions of the multi-model ensemble. The failure probability predicted for each ROI of each case in the **(A)** development and **(B)** held-out test sets is mapped out. The low and high likelihood of being failure are represented in green and red, respectively. **(C)** An example case from the held-out test set is used to demonstrate the multi-model ensemble. Each block composed of 11 × 10 grids represents a single case, corresponding to the outputs from 10 sub-models applied on 11 ROIs. Each sub-model independently generates a prediction on each ROI, and all 11 × 10 outputs are ensembled to reflect the discrepancy among all sub-models. **(D)** Representative attention maps are generated by 10 sub-models independently on the same ROI, providing the failure probabilities and dominant morphology in the process of a comprehensive evaluation. Image size: 50 × 50 μm^2^.

In addition to the individual model, we employed the multi-model ensemble to mimic multiple human experts for consultation, and each sub-model served as an expert with different background. This method allowed us to verify the predictions made by different models trained on different datasets (Table 1). We divided 104 cases in the development set into five portions to generate 10 independent sub-models, and each portion was used for validation (in orange) four times in the development (Figure 1D). Thus, each case included 11 × 4 grids in Figure 6A. We further implemented these 10 sub-models into the held-out test set (Figure 6B), generating 11 × 10 grids for each case to assess the injured regions on 11 ROIs (Figure 6C). We mapped out the integrative reports of all 209 cases in Figure 6, accentuating the individual prediction of each ROI in each case from all available models. In both development and test sets, most non-failure cases had low failure risk predictions (in green) and vice versa (in red). Besides the predicted likelihood of being a failure, our model could simultaneously generate multiple attention maps, providing more intuitive evidence for further predictive decisions on ambiguous cases (Figure 6D).

**Figure 7:**
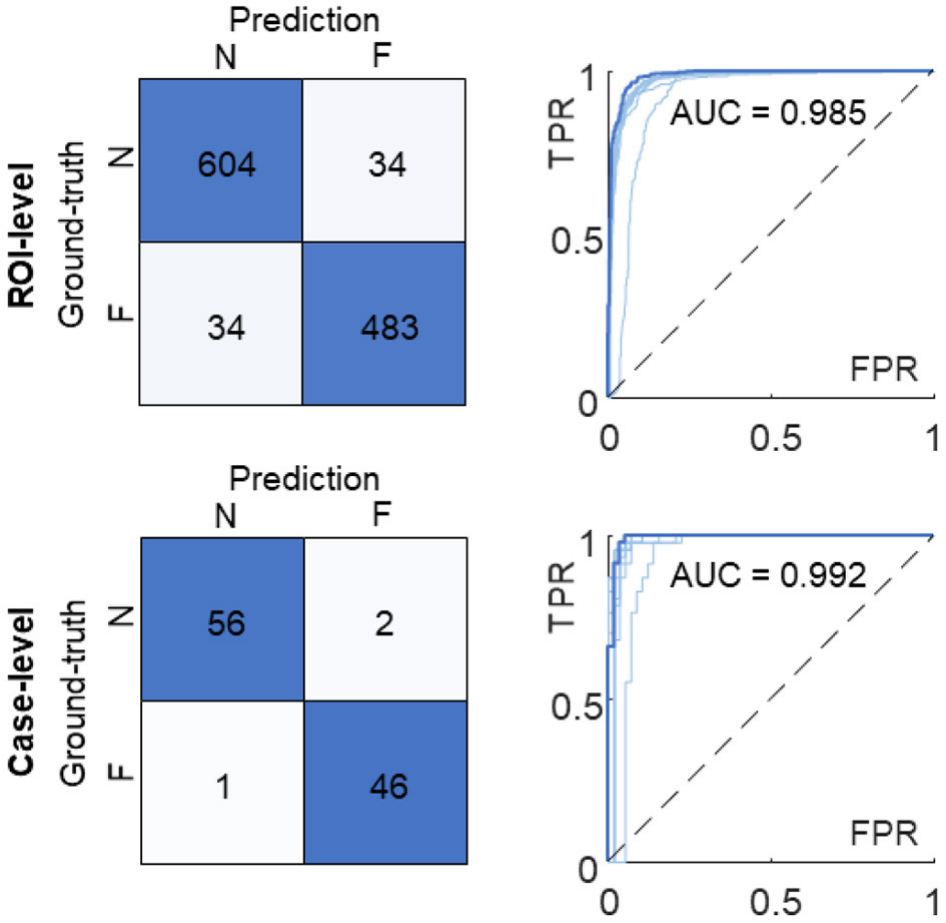
Confusion matrices and ROC curves of the multi-model ensemble. The ROI-level and case-level results are reported at the top and bottom, respectively. The ROC curve and AUC value of the 10-model ensemble (in dark blue) are compared with the ones of individual sub-models (in light blue). N, non-failure; F, failure; ROC, receiver operating characteristic; AUC, area under ROC curve; FPR, false positive rate; TPR, true positive rate.

We averaged corresponding likelihood scores to quantify the prediction on the ROI- and case-level. In comparison with the results of the individual model (Figure 3B), the multi-model ensemble performed 604 and 483 correct classifications on the ROI-level, and detected 56 true non-failure and 46 true failure cases on the case-level (Figure 7). The AUC values of the ensemble model were 0.985 and 0.992 on the ROI- and case-level, respectively, exceeding the average of the 10 sub-models [AUC = 0.971 ± 0.017 and 0.981 ± 0.020 (mean ± standard deviation), respectively], and the AUC values achieved by random forest (AUC = 0.933 and 0.952), and two pathologists (AUC = 0.75, 0.73, on case-level) (23). The quantitative results demonstrated that our multi-model ensemble reduced the misclassification rate, especially on the non-failure cases, and improved the AUC values on both ROI- and case-levels, suggesting its ability to serve as a great complimentary tool to assist clinical diagnosis.

## Discussions

While the clinical diagnosis of heart failure relies on ejection fraction and serum biomarker, EMB is always a useful method for making a definite diagnosis in diseases that are difficult to diagnose by imaging modality alone. Computational pathology techniques based on the deep learning method can reduce the workload on pathologists, particularly for regions that have shortages in access to pathological diagnosis services. Nevertheless, the interpretability issue affects people's trust in deep learning systems. This study established and validated an interpretable DNN model to assess EMB histopathology in response to myocardial injury.

We demonstrated that the well-trained VGG-19 network could distinguish heart failure cases from the non-failure ones using local ROIs selected on whole-slide images. Different from traditional methods that rely on explicit pathological patterns such as cell types (28) or morphological features (29), our model was trained with failure or non-failure label solely. However, the attention maps generated by Grad-CAM were well-localized with representative morphologies such as enlarged nuclei and irregular shapes of cells, indicating that the extracted features from our model pertain to a certain level of pathological knowledge. Combined with Grad-CAM and UMAP embedding methods, we further provided an intuitive visualization of the local and global feature patterns of all EMB images learned by the VGG-19 model. Unlike other applications in cancer (24, 30–32), the implementation of this new model in myocardial injury reveals ill-defined histopathological patterns in local regions, providing a guideline and attention maps for well-trained pathologists. Therefore, integrating VGG-19 with Grad-CAM and UMAP embedding methods provides an interpretive DNN model for more accurate histopathological analyses.

**Figure 8:**
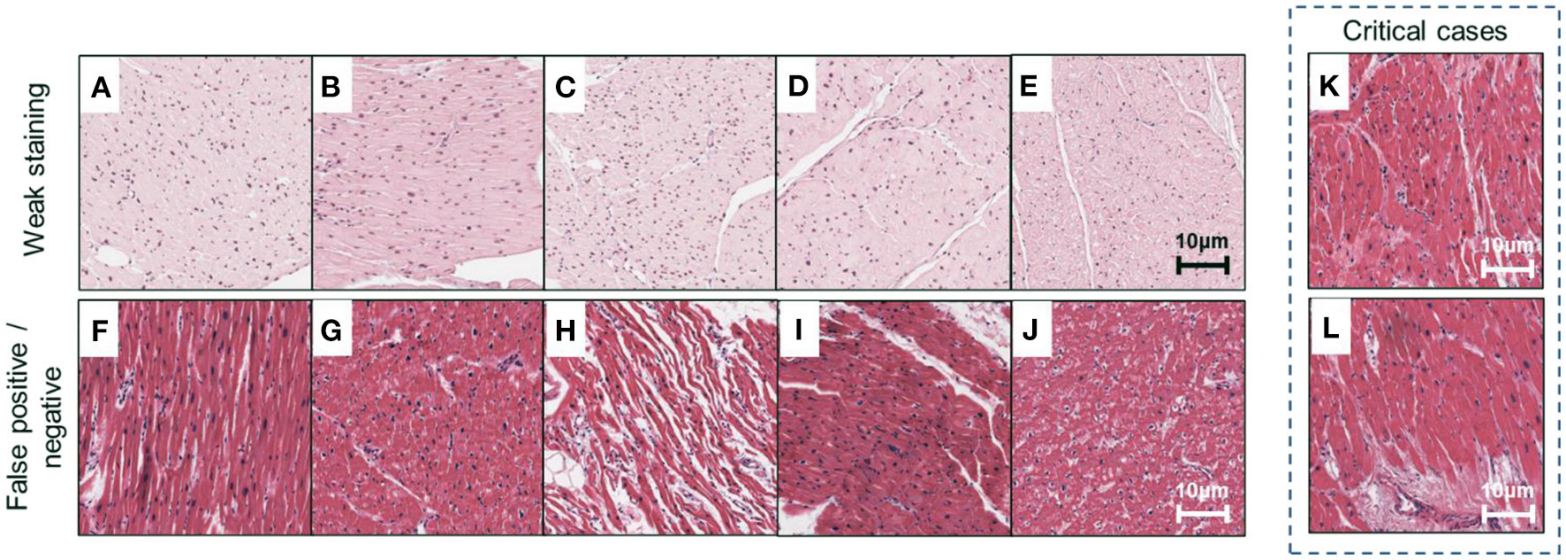
Examples of misclassified cases. Representative ROIs are used as examples to demonstrate two types of misclassification marked in Figure 6. **(A–E)** Weak staining; **(F–I)** false-positive cases; **(J)** false-negative cases; **(K,L)** critical cases are correctly classified but confirmed to exhibit severe local injury and receive high-risk scores. Scale bars: 10 μm.

Our method can be used to obtain the predictive results of each ROI from all ensembled sub-models, leading to an intuitive illustration of the discrepancy among individual sub-models (Figure 8, corresponding to representative results in Figure 6). In this study, we emphasized two types of disagreements among sub-models: (1) a significantly different prediction generated by a sub-model (Figures 8A–E, corresponding to A–E in Figure 6), and (2) in distinguishing false positive or false negative results (Figures 8F–J, corresponding to F–J in Figure 6). The former disagreement is due to the varied staining appearance of specific cases (Figures 8A–E), resulting in an incomprehensive training dataset. Specifically, the data distribution should be inspected prior to model development in response to negative effects introduced by the domain shift (33). The cross-validation protocol employed in this study provides a way to observe such effects in the training dataset. The latter type of disagreement (Figures 8F–J) pertains to transitional predictions, an ambiguous case-related false-positive or false-negative result. In some cases, with the label of “non-failure” (e.g., Figures 8K,L, corresponding to K and L in Figure 6), a few ROIs receive high-risk scores. While the case-level predictions match the ground truth, such circumstance indicates that the case may exhibit severe local injury. To address this issue, we will gather more representative samples, investigate the whole-slide image instead of some specific ROIs, and incorporate other supplementary approaches such as immunohistochemistry staining and polymerase chain reaction (PCR)-based analysis for a comprehensive assessment.

DNN has been proved as a generalizable tool in assisting cardiovascular disease diagnosis, for example, evaluating cardiac allograft rejection by analyzing histopathological or multiplex immunofluorescence images (4, 34). DNN has also led to breakthroughs in other imaging modalities for cardiovascular diagnosis or research, such as echocardiography, coronary artery calcium scoring, coronary computed tomography angiography, light-sheet microscopy, light-field imaging, etc. (17, 35, 36). Numerous applications such as quantification of receptor status (37), estimation of Ki-67 index (38), or implementation into Ziehl-Neelsen staining (39) and Masson's Trichrome (40) are potentially feasible in cardiovascular studies as well. Besides generic image classification and segmentation, DNN has demonstrated its viability to synthesize pseudo H&E images from Raman spectroscopy and other multi-modality non-linear imaging techniques, augmenting non-invasive and *in vivo* diagnosis (41). Collectively, the proposed framework provides a general pipeline for most of modalities and applications, improving the reliability and credibility of deep learning methods in cardiovascular diagnosis and research.

As a data-driven model, an unbiased and comprehensive training dataset is always preferred in an ideal condition. Our current model can be improved from the following aspects. First, the model was trained supervisory with a case-level label (belonging to failure or non-failure group). While the high attention regions are correlated with pathological patterns, these patterns are not explicitly defined and cannot be quantified by DNNs. We will further introduce additional labels to characterize specific pathological patterns, for example, infiltrated inflammation and myocardial necrosis (12). Second, our data were provided by a single institute. We plan to include more diverse data sources to address the issues of varying data quality, processing protocol, and the equipment used for sample preparation (42). A more robust model covering the sample diversity may further advance future clinical investigations.

## Conclusion

In this study, we integrated the VGG-19 network with Grad-CAM, UMAP, and multi-model ensemble methods for assessing EMB images from heart failure cases, providing an interpretive classification with high efficiency and accuracy. Three strategies, including the attention maps produced by Grad-CAM, the deep feature visualization via UMAP embedding, and multi-model ensemble, facilitated the interpretability of this VGG-19 model and clarified the dominant morphologies of injured regions on EMB images. Both individual model and multi-model ensemble indicated that DNN-aided diagnosis had great potential to recognize cardiomyopathy. Overall, our method established the basis for quantitative computation and intuitive interpretation of EMB images that can advance the applications of deep learning models in cardiac research.

## Data Availability Statement

Publicly available datasets were analyzed in this study. This data can be found at: The image data supporting this study can be found via https://idr.openmicroscopy.org/webclient/?show=project-402.

## Author Contributions

YJ, JY, and YD contributed to the development of the intellectual design of the project. YJ and JY performed the experiments and prepared the manuscript. All authors contributed to the data analysis, manuscript revision, and final approval.

## Funding

This work was supported by NIH R00 HL148493 (YD), NSFC 62103098 (JY), and the University of Texas at Dallas.

## Conflict of Interest

The authors declare that the research was conducted in the absence of any commercial or financial relationships that could be construed as a potential conflict of interest.

## Publisher's Note

All claims expressed in this article are solely those of the authors and do not necessarily represent those of their affiliated organizations, or those of the publisher, the editors and the reviewers. Any product that may be evaluated in this article, or claim that may be made by its manufacturer, is not guaranteed or endorsed by the publisher.
